# Complement C5 Activation during Influenza A Infection in Mice Contributes to Neutrophil Recruitment and Lung Injury

**DOI:** 10.1371/journal.pone.0064443

**Published:** 2013-05-16

**Authors:** Cristiana C. Garcia, Wynne Weston-Davies, Remo C. Russo, Luciana P. Tavares, Milene A. Rachid, José C. Alves-Filho, Alexandre V. Machado, Bernhard Ryffel, Miles A. Nunn, Mauro M. Teixeira

**Affiliations:** 1 Laboratório de Imunofarmacologia, Departamento de Bioquímica e Imunologia, ICB, Universidade Federal de Minas Gerais, Belo Horizonte, Minas Gerais, Brazil; 2 Varleigh Ltd, London, United Kingdom; 3 Departamento de Fisiologia e Biofísica, ICB, Universidade Federal de Minas Gerais, Belo Horizonte, Minas Gerais, Brazil; 4 Departamento de Patologia Geral, ICB, Universidade Federal de Minas Gerais, Belo Horizonte, Minas Gerais, Brazil; 5 Departamento de Farmacologia, Faculdade de Medicina de Ribeirão Preto, Universidade de São Paulo, Ribeirão Preto, São Paulo, Brazil; 6 Centro de Pesquisas René Rachou, Fundação Oswaldo Cruz, Belo Horizonte, Minas Gerais, Brazil; 7 CNRS UMR7355, CNRS and University Orleans, France and IIDMM, University of Cape Town, Cape Town, South Africa; 8 Centre for Ecology and Hydrology, Wallingford, United Kingdom; Centre of Influenza Research, The University of Hong Kong, Hong Kong

## Abstract

Influenza virus A (IAV) causes annual epidemics and intermittent pandemics that affect millions of people worldwide. Potent inflammatory responses are commonly associated with severe cases of IAV infection. The complement system, an important mechanism of innate and humoral immune responses to infections, is activated during primary IAV infection and mediates, in association with natural IgM, viral neutralization by virion aggregation and coating of viral hemmagglutinin. Increased levels of the anaphylatoxin C5a were found in patients fatally infected with the most recent H1N1 pandemic virus. In this study, our aim was to evaluate whether targeting C5 activation alters inflammatory lung injury and viral load in a murine model of IAV infection. To address this question C57Bl/6j mice were infected intranasally with 10^4^ PFU of the mouse adapted Influenza A virus A/WSN/33 (H1N1) or inoculated with PBS (Mock). We demonstrated that C5a is increased in bronchoalveolar lavage fluid (BALF) upon experimental IAV infection. To evaluate the role of C5, we used OmCI, a potent arthropod-derived inhibitor of C5 activation that binds to C5 and prevents release of C5a by complement. OmCI was given daily by intraperitoneal injection from the day of IAV infection until day 5. Treatment with OmCI only partially reduced C5a levels in BALF. However, there was significant inhibition of neutrophil and macrophage infiltration in the airways, Neutrophil Extracellular Traps (NETs) formation, death of leukocytes, lung epithelial injury and overall lung damage induced by the infection. There was no effect on viral load. Taken together, these data suggest that targeting C5 activation with OmCI during IAV infection could be a promising approach to reduce excessive inflammatory reactions associated with the severe forms of IAV infections.

## Introduction

The fast activation of the complement system (CS) after a microorganism infects a potential host is an important step in clearance of many pathogens [1]. On the other hand, anaphylatoxins like C3a and C5a, products of the CS cascade, are commonly involved in exacerbated inflammatory reactions that can cause direct harm to the host following infections [2], [3], [4]. Influenza A virus (IAV), an eight segmented, single stranded, negative-sense RNA virus that belongs to the *Orthomyxoviridae* family, [5], is known to activate the CS [6]. CS activation during infection is thought to be important for an effective immune response and virus neutralization in association with natural IgM antibodies in primary infections [7]. Indeed, IAVs have developed mechanisms of evading CS within the host, as shown by the anti-C1q action of influenza protein M1 [8]. The pathology caused by IAV is due mainly to excessive inflammatory responses that culminate in lung damage and acute respiratory distress [9].

Extensive deposition of C4d was found in lungs of fatal cases of influenza caused by IAV pandemic strains, 2009 H1N1and also 1957 H2N2 [10]. Furthermore, increased levels of C3a and C5a had been found in bronchoalveolar lavage fluid (BALF) of mice infected with highly pathogenic avian Influenza H5N1 but not following seasonal IAV infection [11]. The anaphylatoxin C5a is a strong chemoattractant for neutrophils and activates these cells to generate oxidative burst, release of enzymes and phagocytosis (reviewed by [12]). C5a is also able to activate macrophages and endothelial cells and to promote vascular leakage [12]. All these inflammatory responses in which C5a is involved are important for IAV-associated pneumonia. We and others have previously described a direct relation between severity of inflammation and lethality [13], [14]. In addition, CD8^+^ T cell activation during influenza infection requires C5a that acts as a chemoattractant for T lymphocytes [15], [16]. Increased levels of C5a found in BALF of severe cases of patients infected with IAV reinforce the idea that C5a is involved in influenza induced lung pathology [6]. Therefore, we hypothesized that preventing activation of C5 during IAV infection could decrease the magnitude of inflammation.

The co-evolution of pathogens and their hosts has generated a plethora of molecules that increase pathogen survival [17]. We previously described a 17 kDa lipocalin protein derived from salivary gland of the soft tick *Ornithodoros moubata*, OmCI (also known as coversin and rEV576). OmCI binds to C5 and prevents conformational changes that lead to C5a generation and formation of the membrane attack complex (MAC) [18], [19]. OmCI is also reported to capture the inflammatory mediator leukotriene B4 (LTB4) in a binding pocket and to have a neutralizing effect [20], [21]. The protein OmCI has been proven active against a variety of species (mouse, rat, guinea pig, pig and human) which has enabled it to be studied in a wide range of animal models including asthma, immune complex lung disease [20], coronary and renal ischaemia-reperfusion injury and sepsis (unpublished data) and in models of autoimmune peripheral neuropathy [21], [22], [23]. Phase 1 clinical trials of coversin are currently in progress.

In this study, we used OmCI to investigate the inflammatory response to IAV infection in mice and demonstrate that reducing C5 activation decreases IAV mediated inflammation and pathology without increasing viral titer within the lung.

## Materials and Methods

### Ethics Statement

All animal experiments were approved by CETEA/UFMG animal ethics committee (203/08), according to Brazilian national guidelines on animal work.

### Virus

The mouse adapted virus Influenza A/WSN/33 (H1N1)- herein named WSN - was used to infect mice as previously described [13]. Stock virus was produced in chicken eggs and passed once again in eggs and then cultured at a m.o.i of 0.001 in MDCK (Madin-Darby Canine Kidney) cells grown at 37°C and 5% CO_2_ for 72 hours in complete Dulbeccós modified Eagle Medium (DMEM; SIGMA) with 1 mM sodium pyruvate, 4.5 mg/ml L-glucose, 100 U/ml penicillin and 100 µg/ml streptomycin, supplemented with 5% heat inactivated fetal calf serum (FCS; CULTILAB; Brazil). Cell culture supernatants were collected and clarified by low speed centrifugation. Virus aliquots were frozen at –80°C until use. Viral stocks were titrated on MDCK cell monolayers in standard plaque assays using agarose overlay in complete DMEM with 2% FCS. Viral inoculum was prepared by diluting the stock virus in sterile phosphate buffered saline (PBS).

### Animal infections, and treatment with OmCI and Zileuton

Male 8–10 weeks old C57BL/6J mice were maintained in pathogen free conditions at Laboratório de Imunofarmacologia (UFMG/Brazil). Prior to infection, mice were anesthetized with ketamine/xylazine (60 mg/kg, 4 mg/kg, respectively; Syntec, Brazil) and then received, via intranasal administration, 10^4^ PFU of Influenza WSN virus in sterile PBS or PBS only (Mock group) in a total volume of 25μL. Infected mice (5 mice per group) were bled and euthanized 1, 3 and 6 days after infection to obtain Bronchoalveolar Lavage Fluid (BALF) and harvest lungs.

In order to evaluate the effects of OmCI during IAV infection, mice were treated 30 minutes prior to infection with 250µg OmCI diluted in 200 µL of PBS via intraperitoneal injection and received further daily injections of 200 µg OmCI between days 1 to 5. Animals in the vehicle group were injected with 200 µL PBS each day. Weight loss was assessed daily. Five mice per group were euthanized 1, 3 and 6 days after infection to assess leukocyte infiltration into the airways and in lungs, levels of inflammatory mediators in lungs or in BALF, cell free double stranded DNA (cfDNA) in BALF, histopathological changes and viral load in lungs.

The 5-lipoxygenase enzyme (5-LOX) inhibitor Zileuton (Zyflo, Cornerstone Therapeutics In, USA) was used to study the role of leukotrienes during IAV infection. 5-LOX is solely responsible for an early step in the synthesis of leukotrienes. A group of three mice received Zileuton from day zero to day five post-infection and another group of five mice received a combination of Zileuton and OmCI in the same period. Zileuton was given at a dose of 30 mg/kg, diluted in 5% absolute ethanol (Synth, Brazil) in a solution of Carboxymethyl Cellulose 0.5% (Synth, Brazil), via oral gavage in 200 µL.

### Bronchoalveolar lavage and tissue extraction

At indicated time points, infected mice were anesthetized with a solution of 150 mg/kg of ketamine and 10 mg/kg of xylazine and blood was collected by the brachial plexus. Blood was allowed to clot and centrifuged, for collection of serum, which was frozen for further analysis. Once death was confirmed, a 1.7 mm catheter was inserted into the trachea and bronchoalveolar lavage (BAL) performed. To this end, the bronchoalveolar compartment was flushed twice with 1 mL aliquots of PBS going back and forth three times to acquire leukocytes recruited to the airways [24]. After centrifugation, the pellet was used to derive total and differential cell counts and for FACS analysis (see below). After BAL and perfusion of lungs with 5 mL of PBS in order to remove circulating blood, the right lobes were removed and frozen for quantification of Myeloperoxidase (MPO) to assess neutrophil infiltration, inflammatory mediators and plaque assay to measure viral load. Left lobes were fixed in formalin for histological examination.

### Measurement of MPO, inflammatory mediators and total protein

Lung tissue (100 µg) was homogenized in PBS containing antiproteases, as previously described [25]. MPO levels in cell pellets were assayed using a solution of 1.6 mM tetramethylbenzidine and 0.1 mM H_2_O_2_ as previously described [25]. Supernatants were used to assess the concentrations of IFN-γ, CXCL1 and C5a by ELISA using DuoSet kits from R&D Systems, in accordance with the manufacturer’s instructions. BALF was also used to quantify C5a, IFN-γ and CXCL1 levels and serum was used to quantify C5a levels using the same ELISA assay. Total protein quantification based on Bradford’s method (Bio-Rad Protein Assay) was performed on BALF to measure protein leakage to airways.

### cfDNA quantification

As an indirect way to assess NETs and cell death, cfDNA was quantified in cell-free BALF using Quant-iT PicoGreen dsDNA quantification kit (Invitrogen) in accordance with the manufacturer’s instructions. Briefly, the BALF samples and a DNA standard curve (from 1 ng/mL to 1 µg/mL) were diluted in TE buffer and then mixed and incubated for 5 minutes with Quant-iT PicoGreen reagent at room temperature before measuring fluorescence (480 nm for excitation and 520 nm for emission). Samples were compared to the standard curve and the results expressed inµg/mL.

### Flow cytometric analysis of leukocyte populations and cell death

Leukocytes collected from airways of non-infected and infected mice at day 6 after IAV infection were used to analyze cell death by Annexin V staining and Propidium Iodide (PI) incorporation (FITC Annexin V apoptosis detection kit I, BD Pharmigen), according to the manufacturer’s instructions. The populations of macrophages, neutrophils and lymphocytes were analyzed by staining with fluorescent monoclonal antibodies against CD3 (PE-Cy7, BD Biosciences, USA), CD4 (APC, BD Biosciences, USA), CD8 (PerCP, Biolegend, USA), F4/80 (PE-Cy7, eBioscience) and GR1 (Alexa 488, Biolegend). Stained cells were acquired in BD LSRFortessa cell analyzer (BD Biosciences, USA) and analyzed using FlowJo software (Tree Star, Inc., USA).

### Histological analysis

Formalin-fixed left lobes of lungs were dehydrated gradually in ethanol, embedded in paraffin, cut into 4µm sections (3 sections per lung), stained with Hematoxylin and Eosin (H&E) and examined under light microscopy by a pathologist blind to the experimental procedure. All 3 sections of all left lobes were examined and an average score assigned to each lung [26]. The maximum score was 23 and the following parameters were evaluated: airway inflammation (4 points), vascular inflammation (4 points) and parenchymal inflammation (5 points), epithelial injury (5 points) and overall neutrophilic infiltration (5 points).

### Plaque assay

Lungs were collected in sterile conditions, weighed and homogenized in PBS. The viral load in lungs were assessed on MDCK cell monolayers, in standard plaque assays using agarose overlay in complete DMEM with 2% FCS. Briefly, MDCK cell monolayers, in 6 well plates, were incubated for 60 minutes with serial 10-fold dilutions of lung homogenates at 37°C and 5% of CO2, when the agarose monolayer was added. After additional 72 hours of incubation, agarose overlays were removed, the cells where fixed in formalin and stained with crystal violet. The viral titer was expressed as plaque forming unit (PFU) per gram of tissue.

### Statistical analysis

All data are presented as the mean ± SEM and were analyzed using One-way analysis of variance followed by Newman-Keuls post-test to compare different groups. Unpaired t test was used to compare two groups. Statistical significance was set as P<0.05 and all graphs and analysis were performed using Graph Pad Prism 4 software.

## Results

### Local complement activation after Influenza virus A (IAV) infection

**Figure 1:**
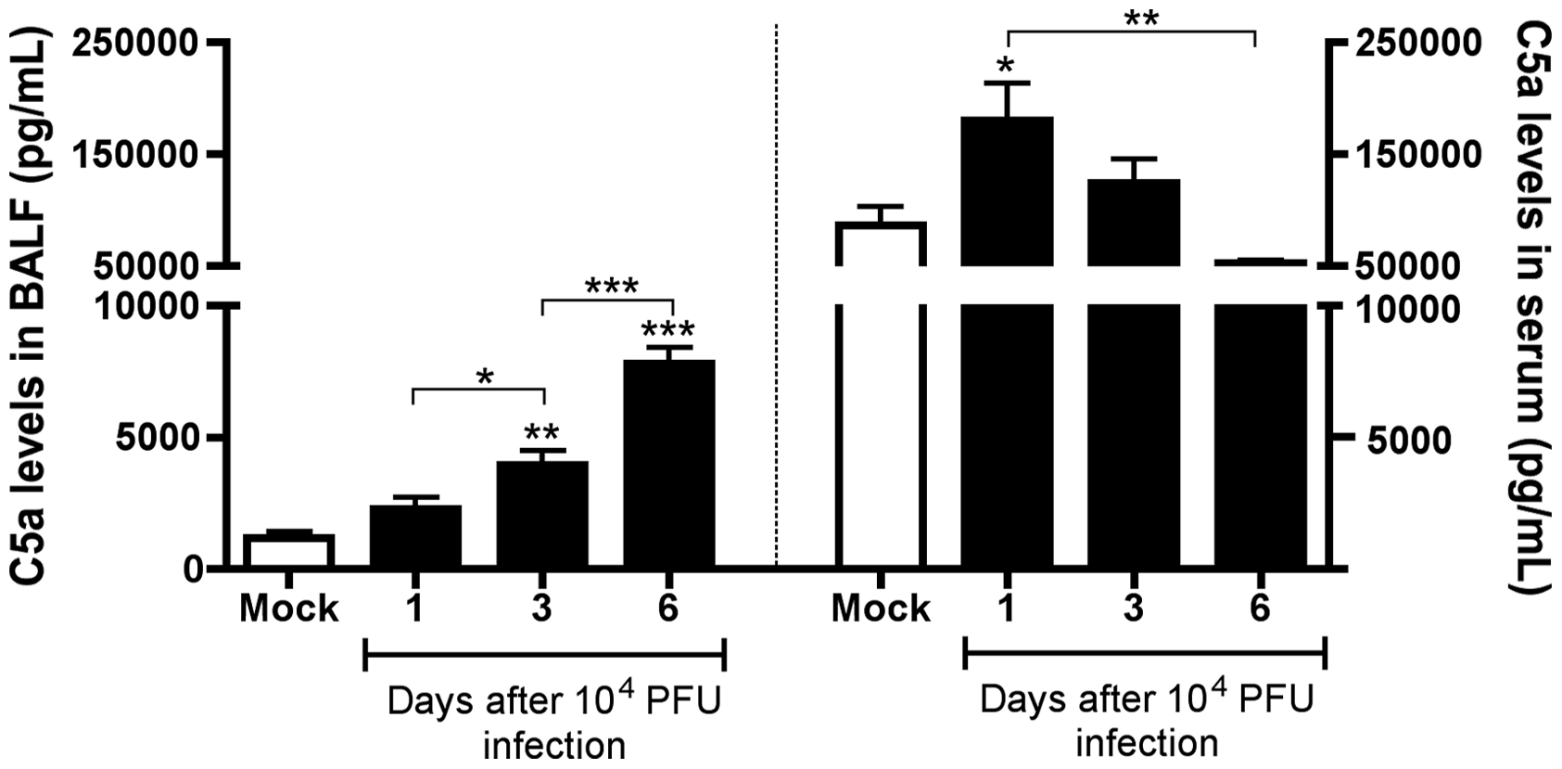
Effects of IAV infection on C5a levels in BALF (left) and serum (right). C57BL/6J mice were infected intranasally with 10^4^ PFU of Influenza A/WSN/33 H1N1, or received PBS intranasally (Mock group). At the indicated time points after infection 5 mice per group were euthanized, blood was collected and BAL performed. Serum and BALF concentrations of C5a were measured by ELISA. Data are presented as Mean ± SEM. *, ** and *** for p<0.05, p<0.01 and p<0.001 respectively, when compared to Mock group or indicated group (One-way ANOVA, Newman-Keuls Multiple Comparison test).

Complement activation following IAV infection has been found to be correlated with clinical disease severity [6], [10], [27]. Thus, aiming to assess complement activation in our system, we infected C57BL6/J mice with 10^4^ PFU of the mouse-adapted Influenza WSN virus. We collected blood, performed bronchoalveolar lavage (BAL) and lungs were harvested 1, 3 and 6 days after infection. We found significantly increased C5a levels in BALF of IAV infected animals from the third to the sixth day of infection, peaking at day 6 after infection (Figure 1, left). By contrast, C5a levels in lung homogenates after infection were similar to mock group at all time points after infection (data not shown), and serum levels of C5a increased at the first day of infection and decreased at day six (Figure 1, right).

### Treatment with OmCI decreases C5a levels, neutrophil and macrophage accumulation and protein leakage after IAV infection

**Figure 2:**
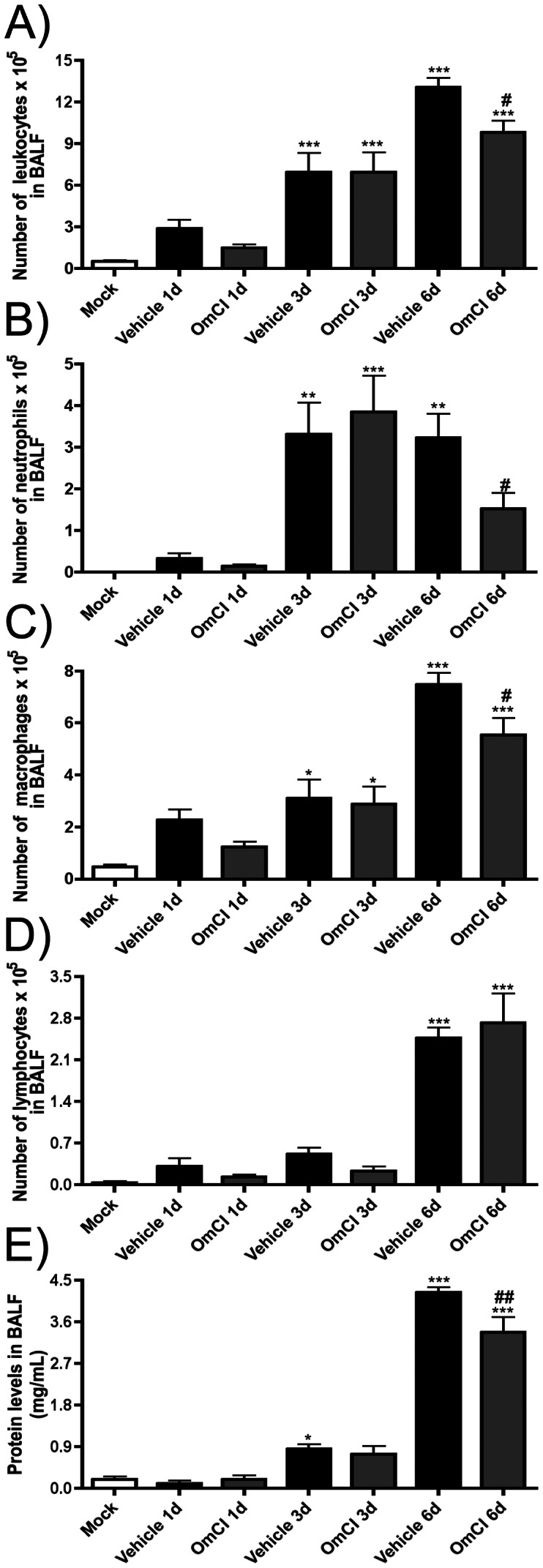
Effects of C5 activation inhibitor OmCI during IAV infection on leukocyte transmigration and protein leakage. C57BL/6J mice were infected intranasally with influenza or Mock infected as described in Figure 1. OmCI treated mice received the protein prior to IAV infection and daily thereafter, while vehicle group received PBS, until one day before the indicated time point. At the 1, 3 and 6 days after infection, mice were euthanized and BAL performed. A) Total number of leukocytes, B) number of neutrophils and C) number of macrophages, D) number of lymphocytes recovered from the airways; E) total protein quantification in BALF. Data are presented as Mean ± SEM. *, ** and *** for p<0.05, p<0.01 and p<0.001 respectively, when compared to Mock group; # and ## for p<0.05, p<0.01 respectively, when compared to Vehicle group sampled on the same day (One-way ANOVA, Newman-Keuls Multiple Comparison test).

**Figure 3:**
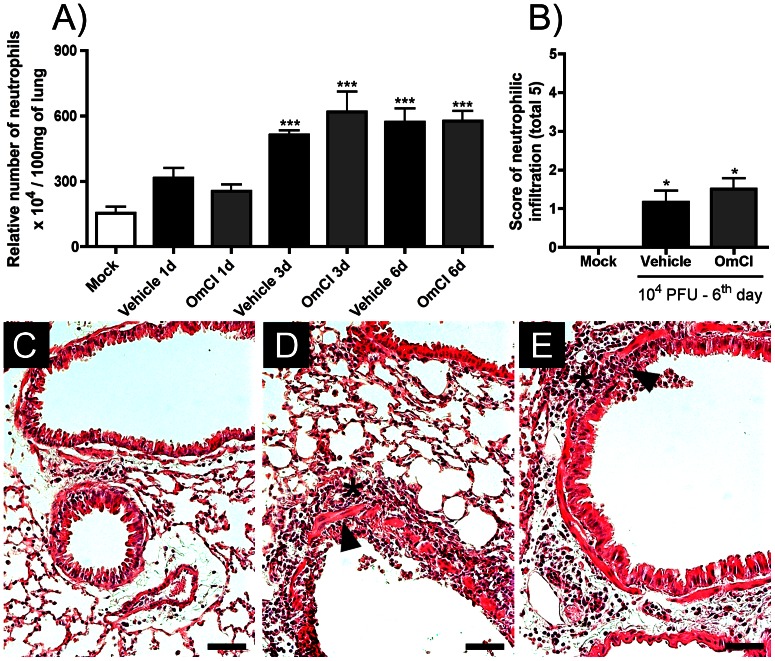
Neutrophil accumulation in lung parenchyma of vehicle and OmCI treated mice. C57BL/6J mice were infected and assigned to treatment groups as in Figure 2. At 1, 3 and 6 days after infection, mice were euthanized, lungs harvested and MPO assayed, to measure neutrophil accumulation in tissue. A) Relative numbers of neutrophils in lungs. At day 6, lungs were harvested for assessment of neutrophil infiltration by analysis of H&E stained lung slides. B) Pathologic score (0–5) of neutrophil accumulation in lungs performed by a pathologist. Representative slides of H&E stained lungs of a C) mock mouse; D) vehicle mouse; E) OmCI treated mouse. Asterisks indicate areas with neutrophils infiltration and arrowheads indicate bronchial epithelial damage. Data are presented as Mean ± SEM. * and *** for p<0.05 and p<0.001, respectively, when compared to Mock group (One-way ANOVA, Newman-Keuls Multiple Comparison test). Bars represent 100 µm.

In order to evaluate a possible role of C5a for the inflammatory responses caused by IAV infection, infected mice were treated with the protein OmCI that binds to C5 and prevents generation of C5a and formation of the membrane attack complex (MAC). Infection with IAV induced an intense influx of leukocytes (Figure 2A) in the airway spaces that peaked at day 6 after infection. The inflammatory infiltrate was predominantly composed of neutrophils (Figure 2B) that were present in high number from the third day after infection and macrophages (Figure 2C) that peaked at day six. Lymphocytes were found in significantly elevated numbers only 6 days after infection (Figure 2D). Protein levels in BALF, a sign of increased vascular permeability [28], were increased from the third day after infection and greatly increased by day 6 (Figure 2E). There was also significant infiltration of neutrophils in lungs of vehicle treated, infected mice as assessed by MPO quantification (Figure 3A) or histopathological analysis (Figure 3B–E; asterisks).

When IAV-infected mice were treated with OmCI, the total number of leukocytes (Figure 2A), neutrophils (Figure 2B) and macrophages (Figure 2C) were reduced in BALF when compared to vehicle treated group at day 6 after infection. This was also confirmed when leukocyte types were evaluated by flow cytometry (data not shown). The number of lymphocytes in BALF of OmCI-treated mice was similar to that of vehicle treated mice (Figure 2D). Protein leakage at day six after IAV infection was reduced in OmCI treated mice, when compared to vehicle group (Figure 2E). Inhibition of neutrophil influx into the airways of OmCI-treated animals was not associated with decrease in accumulation of these cells in lung parenchyma, as assessed by MPO levels (Figure 3A) and histopathological analysis (Figure 3B-E). Both OmCI treated and control mice inoculated with IAV displayed similar weight loss (23.1%±6.2% versus 22.5%±4.6%, respectively).

**Figure 4:**
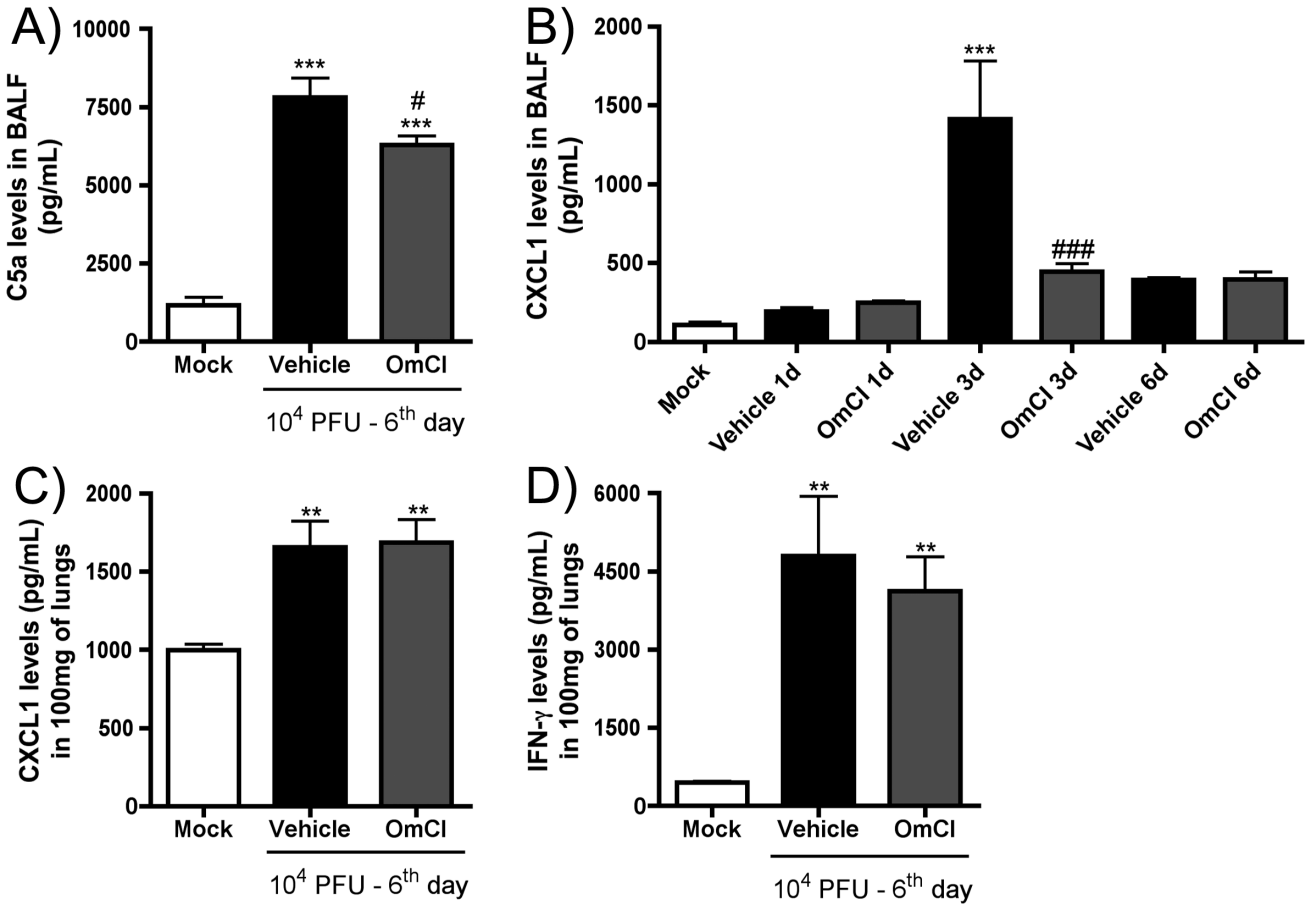
Inflammatory mediator levels after IAV infection. C57BL/6J mice were infected and assigned to treatment groups as in Figure 2. At 1, 3 and 6 days after infection, mice were euthanized, BAL performed and lungs harvested. BALF concentrations of C5a (A), CXCL1 (B) and pulmonary concentrations of CXCL1 (C), and IFN-γ (D) were measured by ELISA. Data are presented as Mean ± SEM. ** and *** for p<0.01 and p<0.001 respectively, when compared to Mock group; # and ### for p<0.05 and p<0.001 respectively, when compared to vehicle group sampled on same day (One-way ANOVA, Newman-Keuls Multiple Comparison test).

IAV infection induced a significant increase in levels of C5a (Figure 4A) and the chemokine CXCL1 (Figure 4B) in BALF and levels of CXCL1 (Figure 4C) and IFN- γ (Figure 4D) in lung homogenates. After OmCI administration into IAV-infected mice, levels of CXCL1 (Figure 4C) and IFN- γ (Figure 4D) were similar to those found in the vehicle group, while C5a levels were partially reduced (Figure 4A). There was very significant inhibition of CXCL1 levels in BALF in OmCI treated mice 3 days after infection (Figure 4B).

### Effects of OmCI treatment on lung damage caused by IAV infection

**Figure 5:**
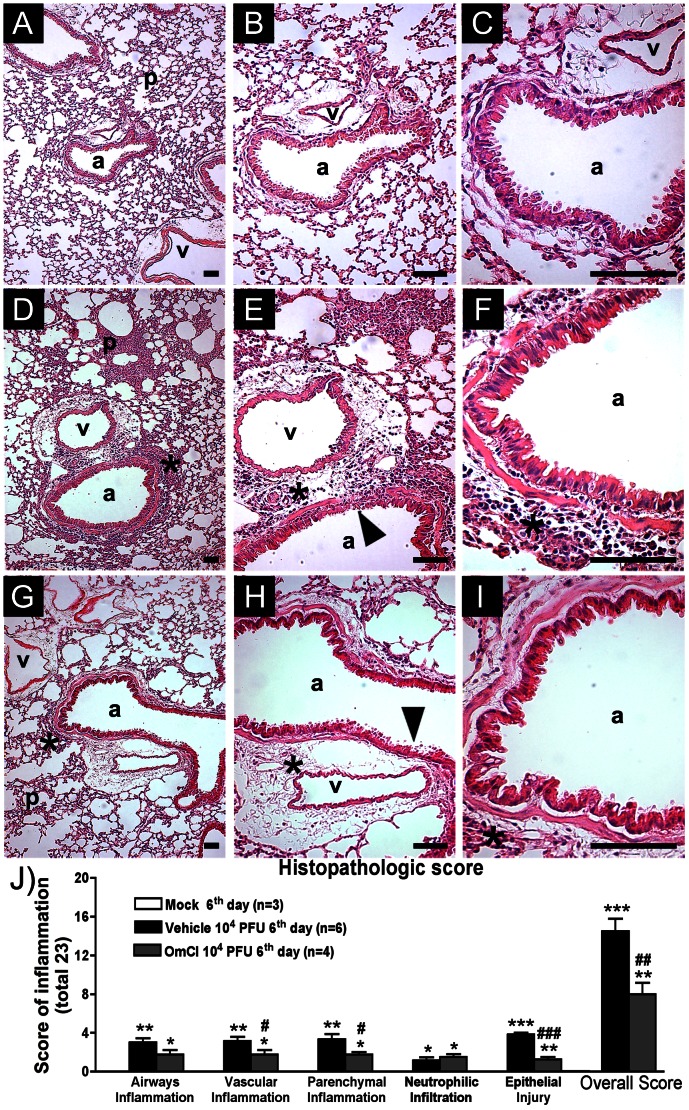
Histopathologic changes caused by IAV are reduced in OmCI treated mice. C57BL/6J mice were infected and assigned to treatment groups as in Figure 2. At the sixth day after infection, mice were euthanized and lungs harvested. Representative slides of H&E stained lungs of mock (A, B, C); vehicle (D, E, F); and OmCI treated mice (G, H, I). “p” represents parenchyma, “v” represents vessels and “a”, airways. Asterisks indicate foci of inflammatory infiltrates and arrowheads indicate areas of epithelial injury. (J) Histopathological score (maximal of 23) evaluated airway, vascular, parenchymal inflammation, neutrophilic infiltration and epithelial injury. Data are presented as Mean ± SEM. *, ** and *** for p<0.05, p<0.01 and p<0.001 respectively, when compared to Mock group; # and ## for p<0.05, p<0.01 respectively, when compared to Vehicle group (One-way ANOVA, Newman-Keuls Multiple Comparison test). Bars represent 100 µm.

Histopathological analysis of lung slides showed that, after 6 days of IAV infection, there was significant inflammatory infiltration in peribronchiolar and perivascular areas in addition to bronchial epithelial damage and parenchymal inflammation. Focal inflammatory infiltrates composed of mononuclear cells and neutrophils were visualized in the septa and alveolar lumen (Figure 5D, E, F) while normal lungs of PBS instilled mice (Mock group) did not present these alterations (Figure 5A, B, C). Remarkably, the overall appearance of the lung of OmCI-treated animals was better than that of vehicle-treated animals and this was reflected in an overall amelioration of pathological scores (Figure 5G, H, I, J). We also used a score system (Figure 5 J) which measures individual airways (a), vascular (v) and parenchymal inflammation (p) as well as neutrophil infiltration (asterisks) and epithelial injury (arrowheads, Figure 3 D, E). In all the evaluated parameters vehicle-treated IAV infected group had a higher score compared to Mock group, but OmCI-treated IAV infected group presented reduced vascular and parenchymal inflammation and also lower epithelial injury. Thus, overall pathologic scores, on a 23 points scale, were reduced in OmCI (8.0±1.1) when compared to vehicle treated group (14.5±1.2), Figure 5 J.

### Effects of OmCI treatment on cfDNA and cell death caused by IAV infection

**Figure 6:**
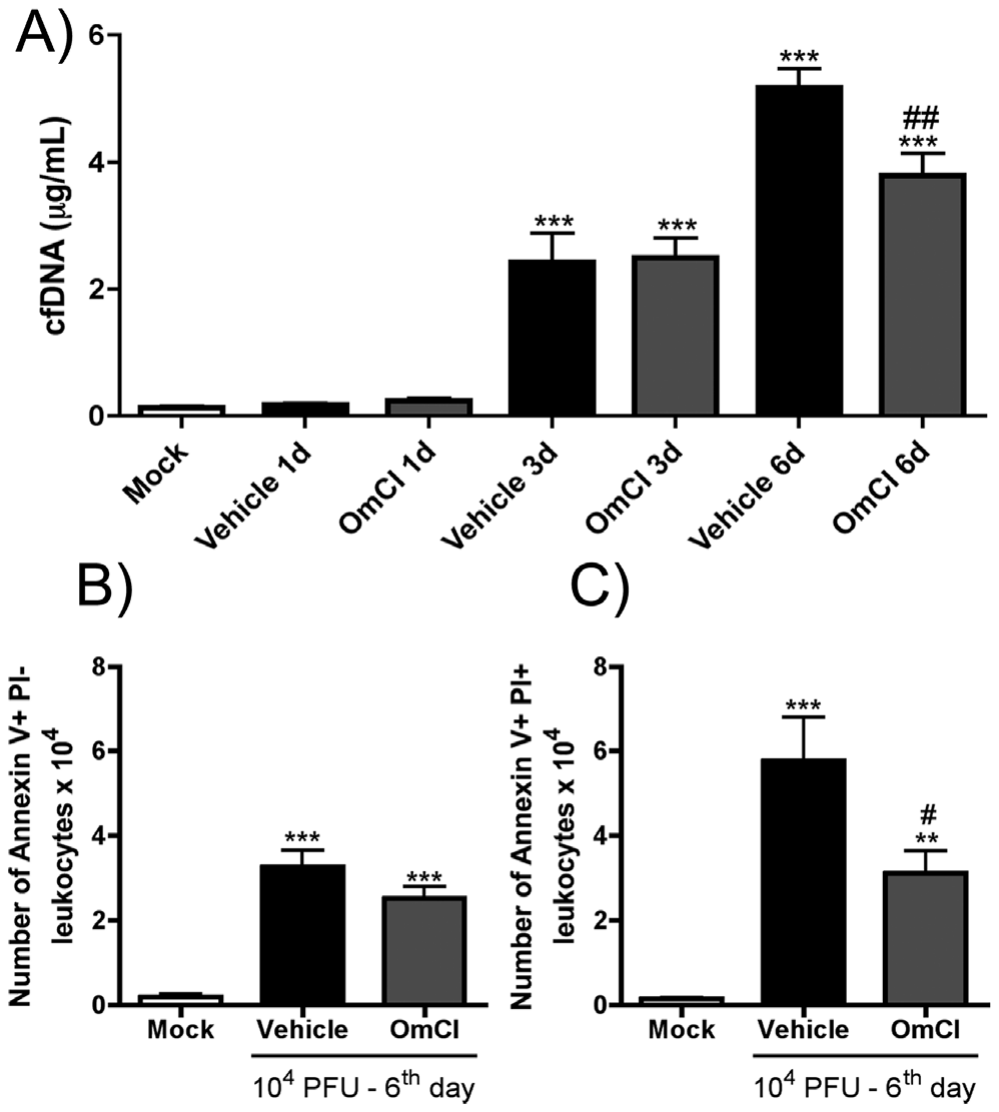
Effects of OmCI on cfDNA levels and number of dead cells in BALF after IAV infection. C57BL/6J mice were infected and assigned to treatment groups as in Figure 2. At1, 3 and 6 days after infection, mice were euthanized and BAL was performed. A) Cell free DNA (cfDNA) levels were measured in BALF by the Quant-iT PicoGreen dsDNA quantification kit. At six days after infection, leukocytes recovered from airways of Mock, Vehicle and OmCI treated mice were analyzed for cellular death by Annexin V and PI incorporation. B) Total apoptotic leukocytes, Annexin V+ PI-, in BALF; C) Total necrotic or late apoptotic leukocytes, Annexin V+ PI+, in BALF. Data are presented as Mean ± SEM. ** and *** for p<0.01 and p<0.001 respectively, when compared to Mock group; # and ## for p<0.05, p<0.01 respectively, when compared to Vehicle group (Kruskal-Wallis test, Dunn’s Multiple Comparison post-test).

NETs are composed of DNA fibers and cytoplasmic proteins and released by neutrophils during infections to kill bacteria, fungi or protozoa [29]. However, NETs may also contribute to tissue damage [30]. The quantification of cell free double-stranded DNA (cfDNA) in BALF is an indirect way to quantify NETs [31] and showed that IAV infection leads to increased cfDNA content from day 3 that continues to increase to day 6 after infection (Figure 6A). Treatment with OmCI was associated with significant reduction in cfDNA levels in BALF at day 6 but not day 3 after infection (Figure 6A).

Cell free DNA can also be found as a result of late apoptosis or necrosis [32]. So, we analyzed whether OmCI treatment affected cell death known to be induced by IAV infection [33]. We analyzed Annexin V binding and PI incorporation in leukocytes recovered from the airways. Indeed, IAV infection caused increased numbers of apoptotic (Annexin V+ PI-, Figure 6B), but also necrotic (or late apoptotic, Annexin V+ PI+, Figure 6C) leukocytes. OmCI treatment resulted in diminished number of necrotic (Figure 6C) but not apoptotic cells (Figure 6B).

### OmCI treatment does not alter the titer of IAV

**Figure 7:**
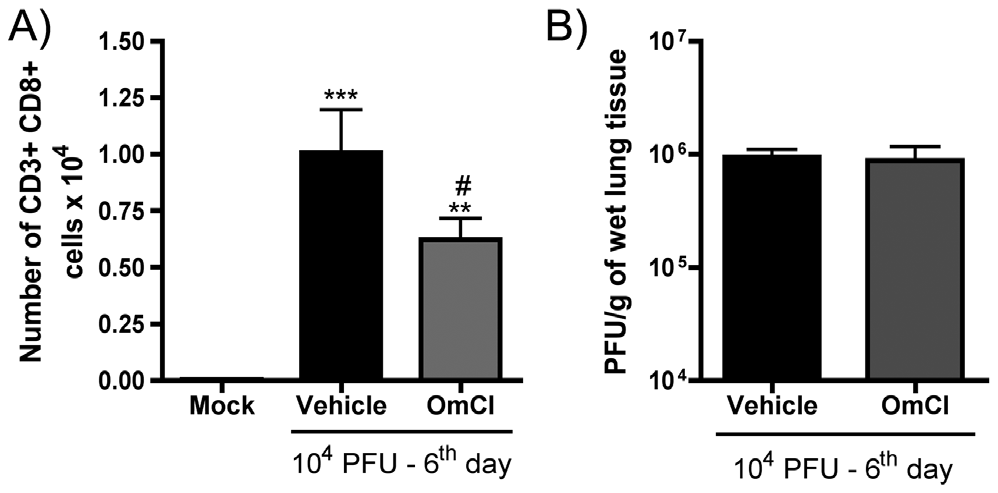
Number of CD8+ T cells and pulmonary viral load after IAV infection and OmCI treatment. C57BL/6J mice were infected and assigned to treatment groups as in Figure 2. A) Numbers of CD3+ CD8+ cells recovered by BAL and analyzed by FACS are reduced in OmCI treated group 6 days after infection. ** and *** for p<0.01 and p<0.001 respectively, when compared to Mock group; # for p<0.05, when compared to Vehicle group (Kruskal-Wallis test, Dunn’s Multiple Comparison post-test). B) Viral titers 6 days after infection in lungs homogenates shown are not changed between vehicle and OmCI treated groups, as assessed by MDCK plaque formation (Unpaired t test).

Complement is a crucial host system that is activated during influenza virus infection and plays an important role in viral clearance [34]. The anaphylatoxin C5a, which is highly chemoattractant to neutrophils [12], is also chemoattractant to T lymphocytes [15] and is important for effective anti-IAV activity mediated by CD8^+^ T cells [35]. Although differential cell counts showed the same number of lymphocytes in vehicle and OmCI treated mice at day 6 after infection (Figure 2 E), further analysis of lymphocyte populations by FACS showed that CD8+ T cells, that are increased in the airways after IAV infection are present in lower numbers in OmCI treated mice (Figure 7A). This difference is not found in CD4+ T cells subset (data not shown). However, despite reduction in CD8+ T cells, viral titers in lungs of mice infected with IAV was not altered by treatment with OmCI (Figure 7B), which suggests viral clearance was unaffected by OmCI.

### Treatment with Zileuton, a 5-LO inhibitor, does not alter IAV-associated pulmonary inflammation

**Figure 8:**
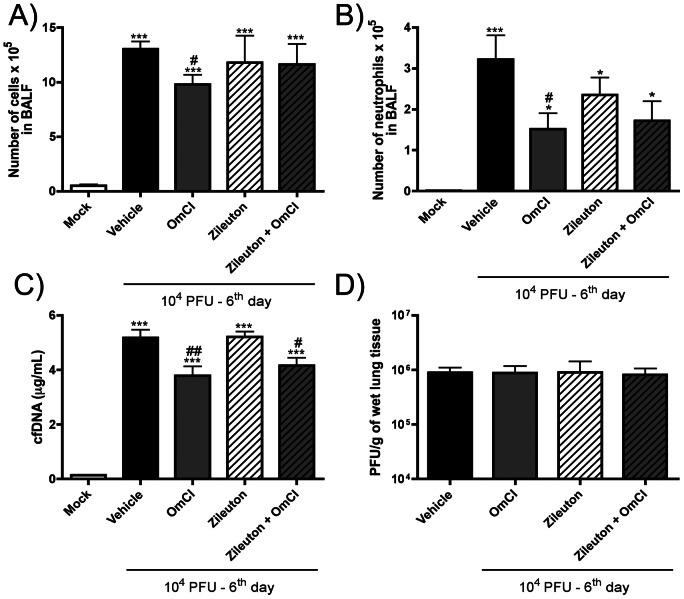
Leukocyte recruitment and cfDNA levels in BALF after IAV infection upon OmCI and Zileuton treatment. C57BL/6J mice were infected intranasally with 10^4^ PFU of Influenza A/WSN/33 H1N1, or received PBS intranasally (Mock group). The 5-LO inhibitor Zileuton (30 mg/kg) was given alone or in combination with OmCI. Mice received the treatment prior to the infection and daily until day 5 after infection, while vehicle group received PBS both, via i.p, Zileuton was given by oral gavage. At the sixth day after infection, mice were euthanized, BAL performed and lungs were harvested. A) Total number of leukocytes; B) number of neutrophils; C) cfDNA levels measured in BALF. Data are presented as Mean ± SEM. * and *** for p<0.05 and p<0.001 respectively, when compared to Mock group; # and ## for p<0.05, p<0.01 respectively, when compared to Vehicle group (One-way ANOVA, Newman-Keuls Multiple Comparison test).

In order to assess whether the LTB4 binding ability of the protein OmCI accounts for its properties during IAV infection, we used Zileuton, a 5-LOX inhibitor, alone or in combination with OmCI. If LTB4 binding by OmCI accounts for the anti-inflammatory activity of OmCI in this model, Zileuton should inhibit IAV associated pulmonary inflammation as effectively as OmCI. After IAV infection, Zileuton treatment or combination of Zileuton and OmCI did not change leukocyte recruitment to the airways (Figure 8A, B) or viral loads (Figure 8D), when compared to vehicle treated mice. The combined treatment of Zileuton and OmCI, but not Zileuton alone, induced similar effects to that of OmCI treatment in cfDNA levels (Figure 8C), which indicates that LTB4 might not be involved in OmCI effects.

## Discussion

Medical concern about IAV pandemics and epidemics faces the challenge of new viral influenza strains that may become resistant to antivirals [36]. For this reason, anti-inflammatory strategies targeting excessive activation of the innate immune system may be a useful adjunctive therapy against IAV infection [13], [37]. We demonstrate here that an arthropod derived inhibitor of C5 activation decreases the recruitment of neutrophils and macrophages to the alveolar space and reduces consequent epithelial damage and lung pathology in IAV infected mice. We also show that reduction of lung inflammation by OmCI did not reduce viral clearance.

A recent paper from O’Brien and colleagues (2011) detected increased C3a and C5a levels in BALF of mice infected with a highly pathogenic H5N1 avian Influenza virus, VN/1194 but not in BALF of animals inoculated with low virulent seasonal or 2009 pandemic viruses [11]. Using the mouse adapted IAV strain A/WSN/33, that mimics a model of severe infection [38] to study innate immune response to the virus we found an increase in C5a levels during the course of infection. O’Brien and colleagues (2011) showed that C3 plays a protective role during IAV infection using a C3 KO mouse strain that showed more severe lung inflammation, weight loss and increased viral load than wild type mice. The protective role of C3 in IAV infection might be explained by the ability of C3a to down-regulate regulatory T cell population and antigen-specific Th17 cells observed in a model of allergic lung inflammation [39]. Furthermore, in a model of IgG immune-complex deposition, C3 deficient mice developed marked lung injury that diminished with C5a blockade [40], suggesting that C5a has a more pathogenic role during inflammation than C3 and its products. This pathogenic role of C5a can be explained by its greater ability to stimulate chemotaxis, granule release and superoxide production in neutrophils than C3a even in low concentrations [41]. The activation of macrophages and endothelial cells by C5a to promote vascular leakage [12], and consequently protein accumulation in the airways, may also explain the protection found after OmCI treatment. Moreover, *in vitro* studies demonstrated that C5a, in association with GM-CSF, is able to induce the release of NETs [42]. It was recently reported that NETs are produced during IAV infection and are associated with alveolar damage in IAV induced pneumonitis [43]. We observed that IAV-induced cfDNA, an index of NETs formation, was reduced after OmCI treatment (Figure 6), and is coincident with reduced lung damage after infection in OmCI treated mice (Figure 5). It is of note that OmCI treatment also reduced number of dead cells in the BAL of infected animals. As dead cells may directly contribute to an increase in cfDNA, the effects of OmCI on cell death may also contribute to the decrease of this parameter, in addition to any possible effect on NET formation.

We found reduced neutrophil and macrophage transmigration and reduced protein leakage in alveolar spaces but no change in the number of neutrophil in lungs of OmCI treated mice (Figures 2 and 3). Accumulation of neutrophils in different lung compartments requires different mechanisms, and the migration of neutrophils to the airways after crossing the epithelial layers is known to be critical for the induction of lung injury [44]. For example, we have previously shown that, in a model of pulmonary fibrosis induced by bleomycin, the blockade of CXCR2, the chemokine receptor involved in neutrophil chemotaxis, led to reduced neutrophil number in BALF and protection from lung fibrosis, but there was still neutrophil accumulation in lungs at a later time point [25]. Blockade of neutrophil and macrophage influx into the airways, accompanied by lower protein leakage by OmCI treatment with consequent reduction in NETs formation, cellular death and epithelial damage may explain the beneficial effects of the protein during IAV infection.

OmCI, also known as coversin and rEV576 is a recombinant small protein belonging to the lipocalin super-family [19] and has a circulating half-life of 30 hours when bound to C5 [18]. It is derived from a native protein discovered in the saliva of the soft tick *Ornothodoros moubata* where its function appears to be to assist the parasite to feed without provoking a host immune response. OmCI has been found to prevent in a dose-related manner the cleavage of complement C5 of all species that it has been tested, including mouse, rat, guinea pig, pig and human [18], [19], [23].

The use of complement antagonists during viral infections faces the challenge of possible impairment of pathogen clearance due to ineffective immune responses against the virus [45]. Reduced levels and activity of specific T CD8 cells were found in mice treated with a C5aR antagonist [35] and C3 deficient mice presented reduced T cell activity and increased viral levels after IAV infection [34]. Nevertheless, here we showed that partially reducing C5a levels through the use of a C5 binding protein during IAV infection indeed reduced CD8+ T cells, but did not affect host response against the virus. Normal viral loads and IFN-γ levels were found in OmCI treated mice, suggesting that the ability of the host to deal with the virus was unaffected. However, inflammation and tissue injury caused by the infection were reduced by OmCI treatment. The concept that there are mediators which contribute to tissue injury but not to host resistance may be useful from a therapeutic point of view [46]. Indeed, our group has provided several proof of concept studies demonstrating the beneficial role of blocking certain aspects of inflammation for the outcome of infection in several animal models, including those caused by bacteria [47], IAV [13], other viruses [48] or protozoan [49].

The molecule CD59a, is a natural regulator of the MAC. It was demonstrated that CD59a KO mice infected with IAV present exacerbated lung inflammation associated with enhanced activation of MAC, excessive neutrophil numbers and death, despite increased virus specific CD4 T cell responses [50]. This latter finding supports our studies by suggesting that excessive C5 activation may cause unwanted inflammation in the context of IAV infection. Furthermore, the latter study suggests that the ability of OmCI to reduce MAC formation [19] may contribute to the observed effects in our model, in addition to an effect on C5a production.

Besides its action in preventing C5 cleavage, OmCI has a neutralizing effect over LTB_4_ [20], [23]. This property of OmCI could potentially contribute to its effects on IAV infection, as LTB4 is a potent neutrophil chemoattractant in its own right. However, treatment of mice with an inhibitor of 5-LO, the enzyme responsible for LTB4 formation, showed that this pathway appears to have little effect in the context of IAV infection. A recent study showed that treatment of mice with a human C5a agonist protected mice from lethality caused by IAV infection by enhancing neutrophil, dendritic cell and NK cell populations [51]. The authors used a small synthetic polypeptide from C5a, called EP67, engineered to eliminate the anaphylactic activity of C5a, because it lacks the ability to bind C5aR (CD88) on neutrophils but can directly activate C5aR on other cells such as APC. Therefore, the authors used a pharmacological strategy to enhance the innate anti-viral immune responses without enhancing viral-associated inflammation. We used a compound which prevents C5a formation, which was associated with decreased neutrophil influx and associated damage. Therefore, although these studies are apparently contradictory, both molecules – EP67 and OmCI – have been engineered to cause reduced inflammation with enhanced (EP67) [51] or unaltered (OmCI) anti-viral immunity. It is possible that the role of complement in preventing excessive inflammation during IAV infection may be virus strain specific or dependent on the particular host. Only studies in humans naturally infected with different strains and subtypes could address this possibility. Notably, other studies using different therapeutic agents have reported benefits associated with decreasing excessive cell influx – macrophages, neutrophils, DCs, CD4 and CD8 T cells – during influenza virus infections (reviewed by [52]). Therefore, the hypothesis that decreasing unwanted inflammation during IAV infection may be beneficial is potentially of interest but needs to be addressed adequately in clinical trials in humans.

The involvement of C5 in many diseases could be studied in a wide range of animal models including asthma, immune complex lung disease, coronary and renal ischemia-reperfusion injury (unpublished data) and in models of autoimmune peripheral neuropathy, using the treatment with OmCI [21], [22]. Phase 1 clinical trials of coversin are currently in progress. If it proves to be safe and effective in blocking the terminal common pathway of the complement cascade in humans there is a possibility that it may prove to be a useful therapeutic agent in the treatment of lung inflammation caused by IAV infection.

## References

[1] Stoermer KA, Morrison TE (2011) Complement and viral pathogenesis. Virology. 10.1016/j.virol.2010.12.045 PMC3073741 21292294

[2] Nascimento EJ, Silva AM, Cordeiro MT, Brito CA, Gil LH, et al (2009) Alternative complement pathway deregulation is correlated with dengue severity. PLoS One 4: e6782. 19707565 10.1371/journal.pone.0006782 PMC2728508

[3] Kanmura S, Uto H, Sato Y, Kumagai K, Sasaki F, et al (2010) The complement component C3a fragment is a potential biomarker for hepatitis C virus-related hepatocellular carcinoma. J Gastroenterol 45: 459–467. 20012107 10.1007/s00535-009-0160-5

[4] Huber-Lang M, Sarma VJ, Lu KT, McGuire SR, Padgaonkar VA, et al (2001) Role of C5a in multiorgan failure during sepsis. J Immunol 166: 1193–1199. 11145701 10.4049/jimmunol.166.2.1193

[5] Bouvier NM, Palese P (2008) The biology of influenza viruses. Vaccine 26 Suppl 4D49–53. 19230160 10.1016/j.vaccine.2008.07.039 PMC3074182

[6] Bjornson AB, Mellencamp MA, Schiff GM (1991) Complement is activated in the upper respiratory tract during influenza virus infection. Am Rev Respir Dis 143: 1062–1066. 2024815 10.1164/ajrccm/143.5_Pt_1.1062

[7] Jayasekera JP, Moseman EA, Carroll MC (2007) Natural antibody and complement mediate neutralization of influenza virus in the absence of prior immunity. J Virol 81: 3487–3494. 17202212 10.1128/JVI.02128-06 PMC1866020

[8] Zhang J, Li G, Liu X, Wang Z, Liu W, et al (2009) Influenza A virus M1 blocks the classical complement pathway through interacting with C1qA. J Gen Virol 90: 2751–2758. 19656971 10.1099/vir.0.014316-0

[9] La Gruta NL, Kedzierska K, Stambas J, Doherty PC (2007) A question of self-preservation: immunopathology in influenza virus infection. Immunol Cell Biol 85: 85–92. 17213831 10.1038/sj.icb.7100026

[10] Monsalvo AC, Batalle JP, Lopez MF, Krause JC, Klemenc J, et al.. (2010) Severe pandemic 2009 H1N1 influenza disease due to pathogenic immune complexes. Nat Med. 10.1038/nm.2262 PMC3034774 21131958

[11] O'Brien KB, Morrison TE, Dundore DY, Heise MT, Schultz-Cherry S (2011) A protective role for complement C3 protein during pandemic 2009 H1N1 and H5N1 influenza A virus infection. PLoS One 6: e17377. 21408070 10.1371/journal.pone.0017377 PMC3052313

[12] Guo RF, Ward PA (2005) Role of C5a in inflammatory responses. Annu Rev Immunol 23: 821–852. 15771587 10.1146/annurev.immunol.23.021704.115835

[13] Garcia CC, Russo RC, Guabiraba R, Fagundes CT, Polidoro RB, et al (2010) Platelet-activating factor receptor plays a role in lung injury and death caused by Influenza A in mice. PLoS Pathog 6: e1001171. 21079759 10.1371/journal.ppat.1001171 PMC2974216

[14] Perrone LA, Plowden JK, Garcia-Sastre A, Katz JM, Tumpey TM (2008) H5N1 and 1918 pandemic influenza virus infection results in early and excessive infiltration of macrophages and neutrophils in the lungs of mice. PLoS Pathog 4: e1000115. 18670648 10.1371/journal.ppat.1000115 PMC2483250

[15] Nataf S, Davoust N, Ames RS, Barnum SR (1999) Human T cells express the C5a receptor and are chemoattracted to C5a. J Immunol 162: 4018–4023. 10201923

[16] Kwan WH, van der Touw W, Paz-Artal E, Li MO, Heeger PS (2013) Signaling through C5a receptor and C3a receptor diminishes function of murine natural regulatory T cells. J Exp Med 210: 257–268. 23382542 10.1084/jem.20121525 PMC3570105

[17] Woolhouse ME, Webster JP, Domingo E, Charlesworth B, Levin BR (2002) Biological and biomedical implications of the co-evolution of pathogens and their hosts. Nat Genet 32: 569–577. 12457190 10.1038/ng1202-569

[18] Hepburn NJ, Williams AS, Nunn MA, Chamberlain-Banoub JC, Hamer J, et al (2007) In vivo characterization and therapeutic efficacy of a C5-specific inhibitor from the soft tick Ornithodoros moubata. J Biol Chem 282: 8292–8299. 17215252 10.1074/jbc.M609858200

[19] Nunn MA, Sharma A, Paesen GC, Adamson S, Lissina O, et al (2005) Complement inhibitor of C5 activation from the soft tick Ornithodoros moubata. J Immunol 174: 2084–2091. 15699138 10.4049/jimmunol.174.4.2084

[20] Roversi P, Ryffel B, Togbe D, Maillet I, Teixeira MM, et al.. (2013) Dual inhibition of Complement C5 and Leukotriene B4 by bifunctional Lipocalin shows both mediators drive murine Immune Complex-induced Acute Lung Injury. (in press). 10.1074/jbc.M112.420331 PMC3696655 23625922

[21] Soltys J, Kusner LL, Young A, Richmonds C, Hatala D, et al (2009) Novel complement inhibitor limits severity of experimentally myasthenia gravis. Ann Neurol 65: 67–75. 19194881 10.1002/ana.21536 PMC3045826

[22] Halstead SK, Humphreys PD, Zitman FM, Hamer J, Plomp JJ, et al (2008) C5 inhibitor rEV576 protects against neural injury in an in vitro mouse model of Miller Fisher syndrome. J Peripher Nerv Syst 13: 228–235. 18844789 10.1111/j.1529-8027.2008.00181.x

[23] Barratt-Due A, Thorgersen EB, Lindstad JK, Pharo A, Lissina O, et al (2011) Ornithodoros moubata complement inhibitor is an equally effective C5 inhibitor in pigs and humans. J Immunol 187: 4913–4919. 21964028 10.4049/jimmunol.1101000 PMC3359935

[24] Narasaraju T, Ng HH, Phoon MC, Chow VT (2010) MCP-1 antibody treatment enhances damage and impedes repair of the alveolar epithelium in influenza pneumonitis. Am J Respir Cell Mol Biol 42: 732–743. 19617401 10.1165/rcmb.2008-0423OC PMC2891499

[25] Russo RC, Guabiraba R, Garcia CC, Barcelos LS, Roffe E, et al (2009) Role of the chemokine receptor CXCR2 in bleomycin-induced pulmonary inflammation and fibrosis. Am J Respir Cell Mol Biol 40: 410–421. 18836137 10.1165/rcmb.2007-0364OC

[26] Horvat JC, Beagley KW, Wade MA, Preston JA, Hansbro NG, et al (2007) Neonatal chlamydial infection induces mixed T-cell responses that drive allergic airway disease. Am J Respir Crit Care Med 176: 556–564. 17600276 10.1164/rccm.200607-1005OC

[27] Ohta R, Torii Y, Imai M, Kimura H, Okada N, et al. Serum concentrations of complement anaphylatoxins and proinflammatory mediators in patients with 2009 H1N1 influenza. Microbiol Immunol. 10.1111/j.1348-0421.2011.00309.x 21244468

[28] Lin KL, Suzuki Y, Nakano H, Ramsburg E, Gunn MD (2008) CCR2+ monocyte-derived dendritic cells and exudate macrophages produce influenza-induced pulmonary immune pathology and mortality. J Immunol 180: 2562–2572. 18250467 10.4049/jimmunol.180.4.2562

[29] Papayannopoulos V, Zychlinsky A (2009) NETs: a new strategy for using old weapons. Trends Immunol 30: 513–521. 19699684 10.1016/j.it.2009.07.011

[30] Logters T, Margraf S, Altrichter J, Cinatl J, Mitzner S, et al (2009) The clinical value of neutrophil extracellular traps. Med Microbiol Immunol 198: 211–219. 19653000 10.1007/s00430-009-0121-x

[31] Douda DN, Jackson R, Grasemann H, Palaniyar N (2011) Innate immune collectin surfactant protein D simultaneously binds both neutrophil extracellular traps and carbohydrate ligands and promotes bacterial trapping. J Immunol 187: 1856–1865. 21724991 10.4049/jimmunol.1004201

[32] Pisetsky DS (2012) The origin and properties of extracellular DNA: from PAMP to DAMP. Clin Immunol 144: 32–40. 22659033 10.1016/j.clim.2012.04.006 PMC3724456

[33] Tran AT, Cortens JP, Du Q, Wilkins JA, Coombs KM (2013) Influenza virus induces apoptosis via BAD-mediated mitochondrial dysregulation. J Virol 87: 1049–1060. 23135712 10.1128/JVI.02017-12 PMC3554053

[34] Kopf M, Abel B, Gallimore A, Carroll M, Bachmann MF (2002) Complement component C3 promotes T-cell priming and lung migration to control acute influenza virus infection. Nat Med 8: 373–378. 11927943 10.1038/nm0402-373

[35] Kim AH, Dimitriou ID, Holland MC, Mastellos D, Mueller YM, et al (2004) Complement C5a receptor is essential for the optimal generation of antiviral CD8+ T cell responses. J Immunol 173: 2524–2529. 15294968 10.4049/jimmunol.173.4.2524

[36] Kiso M, Mitamura K, Sakai-Tagawa Y, Shiraishi K, Kawakami C, et al (2004) Resistant influenza A viruses in children treated with oseltamivir: descriptive study. Lancet 364: 759–765. 15337401 10.1016/S0140-6736(04)16934-1

[37] Zheng BJ, Chan KW, Lin YP, Zhao GY, Chan C, et al (2008) Delayed antiviral plus immunomodulator treatment still reduces mortality in mice infected by high inoculum of influenza A/H5N1 virus. Proc Natl Acad Sci U S A 105: 8091–8096. 18523003 10.1073/pnas.0711942105 PMC2430364

[38] Kobasa D, Takada A, Shinya K, Hatta M, Halfmann P, et al (2004) Enhanced virulence of influenza A viruses with the haemagglutinin of the 1918 pandemic virus. Nature 431: 703–707. 15470432 10.1038/nature02951

[39] Lim H, Kim YU, Drouin SM, Mueller-Ortiz S, Yun K, et al (2012) Negative regulation of pulmonary Th17 responses by C3a anaphylatoxin during allergic inflammation in mice. PLoS One 7: e52666. 23285141 10.1371/journal.pone.0052666 PMC3527591

[40] Huber-Lang M, Sarma JV, Zetoune FS, Rittirsch D, Neff TA, et al (2006) Generation of C5a in the absence of C3: a new complement activation pathway. Nat Med 12: 682–687. 16715088 10.1038/nm1419

[41] Law SKA, Reid KBM (1995) Complement. Oxford: Oxford University Press.

[42] Yousefi S, Mihalache C, Kozlowski E, Schmid I, Simon HU (2009) Viable neutrophils release mitochondrial DNA to form neutrophil extracellular traps. Cell Death Differ 16: 1438–1444. 19609275 10.1038/cdd.2009.96

[43] Narasaraju T, Yang E, Samy RP, Ng HH, Poh WP, et al (2011) Excessive neutrophils and neutrophil extracellular traps contribute to acute lung injury of influenza pneumonitis. Am J Pathol 179: 199–210. 21703402 10.1016/j.ajpath.2011.03.013 PMC3123873

[44] Li Q, Park PW, Wilson CL, Parks WC (2002) Matrilysin shedding of syndecan-1 regulates chemokine mobilization and transepithelial efflux of neutrophils in acute lung injury. Cell 111: 635–646. 12464176 10.1016/s0092-8674(02)01079-6

[45] Dunkelberger JR, Song WC (2010) Role and mechanism of action of complement in regulating T cell immunity. Mol Immunol 47: 2176–2186. 20603023 10.1016/j.molimm.2010.05.008 PMC2923545

[46] Garcia CC, Guabiraba R, Soriani FM, Teixeira MM (2010) The development of anti-inflammatory drugs for infectious diseases. Discov Med 10: 479–488. 21189219

[47] Martin EL, Souza DG, Fagundes CT, Amaral FA, Assenzio B, et al (2010) Phosphoinositide-3 kinase gamma activity contributes to sepsis and organ damage by altering neutrophil recruitment. Am J Respir Crit Care Med 182: 762–773. 20508212 10.1164/rccm.201001-0088OC

[48] Souza DG, Fagundes CT, Sousa LP, Amaral FA, Souza RS, et al (2009) Essential role of platelet-activating factor receptor in the pathogenesis of Dengue virus infection. Proc Natl Acad Sci U S A 106: 14138–14143. 19666557 10.1073/pnas.0906467106 PMC2729033

[49] Lacerda-Queiroz N, Rodrigues DH, Vilela MC, Rachid MA, Soriani FM, et al (2012) Platelet-activating factor receptor is essential for the development of experimental cerebral malaria. Am J Pathol 180: 246–255. 22079430 10.1016/j.ajpath.2011.09.038

[50] Longhi MP, Williams A, Wise M, Morgan BP, Gallimore A (2007) CD59a deficiency exacerbates influenza-induced lung inflammation through complement-dependent and -independent mechanisms. Eur J Immunol 37: 1266–1274. 17429844 10.1002/eji.200636755 PMC2435422

[51] Sanderson SD, Thoman ML, Kis K, Virts EL, Herrera EB, et al (2012) Innate immune induction and influenza protection elicited by a response-selective agonist of human c5a. PLoS One 7: e40303. 22792270 10.1371/journal.pone.0040303 PMC3391237

[52] Damjanovic D, Small CL, Jeyananthan M, McCormick S, Xing Z (2012) Immunopathology in influenza virus infection: uncoupling the friend from foe. Clin Immunol 144: 57–69. 22673491 10.1016/j.clim.2012.05.005

